# Methods of Blinding in Reports of Randomized Controlled Trials Assessing Pharmacologic Treatments: A Systematic Review

**DOI:** 10.1371/journal.pmed.0030425

**Published:** 2006-10-31

**Authors:** Isabelle Boutron, Candice Estellat, Lydia Guittet, Agnes Dechartres, David L Sackett, Asbjørn Hróbjartsson, Philippe Ravaud

**Affiliations:** 1 INSERM U738, Paris, France; 2 Université Paris 7, UFR de Médecine, Paris, France; 3 AP-HP, Hópital Bichat, Département d'Epidémiologie, Biostatistique et Recherche Clinique, Paris, France; 4 Trout Research and Education Centre at Irish Lake, Markdale, Ontario, Canada; 5 Nordic Cochrane Centre, Rigshospitalet, Copenhagen, Denmark; GlaxoSmithKline, United Kingdom

## Abstract

**Background:**

Blinding is a cornerstone of therapeutic evaluation because lack of blinding can bias treatment effect estimates. An inventory of the blinding methods would help trialists conduct high-quality clinical trials and readers appraise the quality of results of published trials. We aimed to systematically classify and describe methods to establish and maintain blinding of patients and health care providers and methods to obtain blinding of outcome assessors in randomized controlled trials of pharmacologic treatments.

**Methods and Findings:**

We undertook a systematic review of all reports of randomized controlled trials assessing pharmacologic treatments with blinding published in 2004 in high impact-factor journals from Medline and the Cochrane Methodology Register. We used a standardized data collection form to extract data. The blinding methods were classified according to whether they primarily (1) established blinding of patients or health care providers, (2) maintained the blinding of patients or health care providers, and (3) obtained blinding of assessors of the main outcomes. We identified 819 articles, with 472 (58%) describing the method of blinding. Methods to establish blinding of patients and/or health care providers concerned mainly treatments provided in identical form, specific methods to mask some characteristics of the treatments (e.g., added flavor or opaque coverage), or use of double dummy procedures or simulation of an injection. Methods to avoid unblinding of patients and/or health care providers involved use of active placebo, centralized assessment of side effects, patients informed only in part about the potential side effects of each treatment, centralized adapted dosage, or provision of sham results of complementary investigations. The methods reported for blinding outcome assessors mainly relied on a centralized assessment of complementary investigations, clinical examination (i.e., use of video, audiotape, or photography), or adjudication of clinical events.

**Conclusions:**

This review classifies blinding methods and provides a detailed description of methods that could help trialists overcome some barriers to blinding in clinical trials and readers interpret the quality of pharmalogic trials.

## Abstract 2

Following a systematic review of all reports of randomized controlled trials assessing pharmacologic treatments involving blinding, a classification of blinding methods is proposed.

## Editors' Summary

**Background.:**

In evidence-based medicine, good-quality randomized controlled trials are generally considered to be the most reliable source of information about the effects of different treatments, such as drugs. In a randomized trial, patients are assigned to receive one treatment or another by the play of chance. This technique helps makes sure that the two groups of patients receiving the different treatments are equivalent at the start of the trial. Proper randomization also prevents doctors from controlling or affecting which treatment patients get, which could distort the results. An additional tool that is also used to make trials more precise is “blinding.” Blinding involves taking steps to prevent patients, doctors, or other people involved in the trial (e.g., those people recording measurements) from finding out which patients got what treatment. Properly done, blinding should make sure the results of a trial are more accurate. This is because in an unblinded study, participants may respond better if they know they have received a promising new treatment (or worse if they only got placebo or an old drug); doctors may “want” a particular treatment to do better in the trial, and unthinking bias could creep into their measurements or actions; the same applies for practitioners and researchers who record patients' outcomes in the trial. However, blinding is not a simple, single step; the people carrying out the trial often have to set up a variety of different procedures that depend on the type of trial that is being done.

**Why Was This Study Done?:**

The researchers here wanted to thoroughly examine different methods that have been used to achieve blinding in randomized trials of drug treatments, and to describe and classify them. They hoped that a better understanding of the different blinding methods would help people doing trials to design better trials in the future, and also help readers to interpret the quality of trials that had been done.

**What Did the Researchers Do and Find?:**

This group of researchers conducted what is called a “systematic review.” They systematically searched the published medical literature to find all randomized, blinded drug trials published in 2004 in a number of different “high-impact” journals (journals whose articles are often mentioned in other articles). Then, the researchers classified information from the published trial reports. The researchers ended up with 819 trial reports, and nearly 60% of them described how blinding was done. Their classification of blinding was divided up into three main areas. First, they detailed methods used to hide which drugs are given to particular patients, such as preparing identically appearing treatments; using strong flavors to mask taste; matching the colors of pills; using saline injections and so on. Second, they described a number of methods that could be used to reduce the risk of unblinding (of doctors or patients), such as using an “active placebo” (a sugar pill that mimics some of the expected side effects of the drug treatment). Finally, they defined methods for blinded measurement of outcomes (such as using a central committee to collect data).

**What Do These Findings Mean?:**

The researchers' classification will help people to work out how different techniques can be used to achieve, and keep, blinding in a trial. This will assist others to understand whether any particular trial was likely to have been blinded properly, and therefore work out whether the results are reliable. The researchers also suggest that, generally, blinding methods are not described in enough detail in published scientific papers, and recommend that guidelines for describing results of randomized trials be improved.

**Additional Information.:**

Please access these Web sites via the online version of this summary at http://dx.doi.org/10.1371/journal.pmed.0030425.

- James Lind Library has been created to help patients and researchers understand fair tests of treatments in health care by illustrating how fair tests have developed over the centuries
- ClinicalTrials.gov, a trial registry created by the US National Institutes of Health, has an introduction to understanding clinical trials
- National Electronic Library for Health introduction to controlled clinical trials

## Introduction

Randomized controlled trials (RCTs) are widely recognized as the criterion standard for unbiased assessment of the effect of different pharmacologic treatments. Blinding is used in combination with randomization to limit the occurrence of conscious and unconscious bias in the conduct of clinical trials (performance bias) and interpretation of outcomes (ascertainment bias). Blinding is not well understood. For example, it has been demonstrated that the terms single and double blind frequently used by researchers and widely accepted by readers as a key marker of validity of an RCT lack consistency in use and interpretation [1–3]. In clinical trials, blinding in RCTs refers to keeping study participants, health care providers, and those assessing outcomes unaware of the assigned intervention. Empirical evidence suggests that lack of blinding involves inflated treatment effect estimates [2,4–6]. In fact, blinded patients may report symptoms differently from unblinded patients or have different thresholds for leaving a trial or seeking additional treatment outside a trial. Similarly, lack of blinding of health care providers can result in systematic differences in care provided apart from the intervention being evaluated (performance bias). Furthermore, lack of blinding of outcome assessors can result in systematic differences in outcome assessment (ascertainment bias) [7,8]. Blinding is particularly important when outcome measures involve some subjectivity, such as assessment of pain or cause of death. Blinding is probably less important for more objective outcomes such as death, because the risk of ascertainment bias is limited. An example of ascertainment bias was seen in a multiple sclerosis trial in which assessment by unblinded neurologists demonstrated an apparent treatment benefit, whereas that by blinded neurologists did not [9].

Blinding of patients and health care providers can be difficult to establish at the start of a trial and to maintain during a trial. For example, unblinding may occur during the trial for patients and health care providers because of specific side effects or specific requirements such as dosage modification [10,11]. Furthermore, obtaining blinded outcome assessment in clinical trials is difficult, for example, if patients are not blinded and could reveal their treatment to the outcome assessor. Whether these problems are overcome depend in part on the quality and creativity of the blinding methods.

To our knowledge, a detailed description of blinding methods has never been published. Thus, we aimed to describe and classify methods used for blinding in RCTs assessing pharmacologic treatment. This classification should help in the dissemination of these methods for trialists who perform RCTs and also the assessment of the quality of results of clinical trials for readers.

## Methods

We systematically summarized and categorized the method of blinding patients, health care providers, and outcome assessors in RCTs of pharmacologic treatment published in 2004 in high impact-factor journals.

### Search Strategy and Selection of Reports

We identified reports of all RCTs published in 2004 in high impact-factor journals (three highest impact factors for each subject category of the Journal Citation Reports 2003 [12], such as cardiac and cardiovascular system, respiratory system or rheumatology, and ten of the highest-impact general medical journals) and indexed in Medline by searching PubMed (http://www.ncbi.nlm.nih.gov/entrez/query.fcgi?db=PubMed) using the terms “double-blind method” [MESH term] OR “single-blind method” [MESH term] limited to RCTs. This search strategy might have missed some articles describing a blinded method; however, our aim was not to be exhaustive but to provide a description of blinding methods and to propose a useful classification.

One of us (IB) assessed retrieved reports by screening the titles and abstracts to identify the relevant studies. We included reports only if the study design was identified as an RCT assessing pharmacologic treatments and published as a full-text article. We excluded nonrandomized trials, pharmacokinetic studies, follow-up trials, diagnostic assessment, pathophysiologic studies, and ancillary studies from an RCT for subgroup analysis, cost-effectiveness trials, meta-analyses, and trials assessing nonpharmacologic treatments. After obtaining the full text, we selected reports only if they reported that there was blinding of at least one of the following: patients, health care providers, or outcome assessors.

We furthermore searched for reports on blinding indexed in the Cochrane Methodology Register (http://www.cochrane.org/access_data/cmr/accessDB_cmr.asp) using the term “blind.” We included reports if, in the title or abstract, authors reported the use of a specific method of blinding or a specific placebo. We gathered additional relevant trials by searching references of relevant selected reports or those known by members of our team or experts in this field.

### Data Extraction

The research team compiled a standardized data collection form. Before data extraction, as a planned calibration exercise, four members of the team (IB, CE, LG, and AD) independently evaluated a separate set of ten reports. At a meeting, the group reviewed evaluations and resolved any disagreements by consensus. We randomly allocated the selected articles to each reviewer, who independently completed the data extraction for one-quarter of the articles selected. Reviewers assessed the title, abstract, methods, and results sections.

We obtained data on the description of the experimental treatment (oral drug, intramuscular, intravenous, intra-articular treatment, or other) and the control treatment (placebo, active control treatment, or usual care). Reviewers also identified and classified the primary outcome according to specific criteria used previously [13] as (1) a patient-reported outcome, whereby the patient is the outcome assessor (e.g., pain, disability, quality of life); (2) physician-driven data that suppose a contact between patients and outcome assessors (e.g., range of motion); (3) complementary investigations that do not suppose a contact between patients and outcome assessors (e.g., international normalization ratios within the target range, angiographic restenosis); or (4) a clinical event determined by the interaction between patients and care providers (e.g., death, myocardial infarction, stroke, dialysis, or blood transfusion). Reviewers extracted the reported blinding status for patients, health care providers, and outcome assessors and checked whether the methods of blinding were described and how. For this purpose, reviewers checked for the reporting of a method to establish blinding for patients and/or health care providers—that is, methods to provide indistinguishable treatments in each arm such as use of matching placebo, bottle covered with an opaque bag, or use of a double dummy. Reviewers also checked for the reporting of a procedure to avoid unblinding of patients and/or care providers because of side effects or specific requirements such as dosage modifications. Finally, we recorded the description of specific methods of blinded outcome assessments such as patients being informed not to tell the outcome assessors what treatment they received or centralized assessment of complementary investigations.

### Classification

We used a simple system to classify the blinding methods according to (1) who was blinded (patient, health care providers, or outcome assessors) and (2) a distinction between establishing and maintaining blinding. Thus, we classified blinding methods according to whether they primarily (1) established blinding of patients or health care providers (through the mode of administration and the physical characteristics of the treatments), (2) maintained the blinding of patients or health care provider (through the risk of unblinding by specific adverse effects or specific requirements such as dosage modifications), or (3) obtained the blinding of assessors (through the category of main outcome, i.e., patient-reported outcome, physician-driven data, complementary investigations, and clinical events).

### Statistical Analysis

We describe descriptive statistics for categorical variables with frequencies and percentages. All data analyses involved use of the SAS system for Windows, Release 9.1 (SAS Institute, Cary, North Carolina, United States).

## Results

### Articles Selected

**Figure 1:**
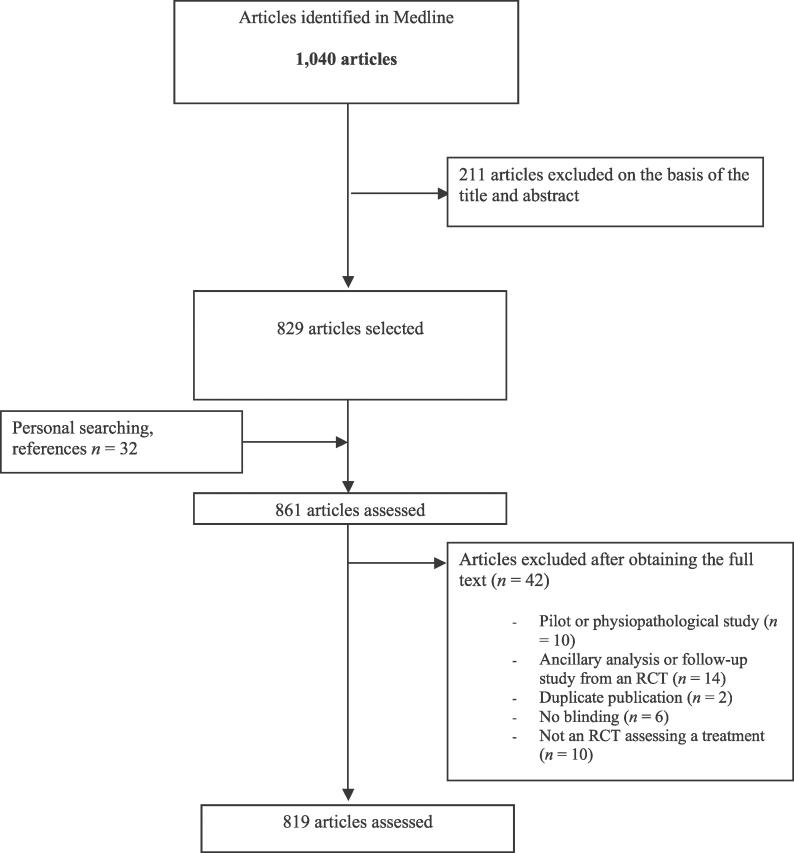
Study Screening Process

We retrieved 1,040 articles by electronic search and excluded 211 reports on the basis of the title and abstract and 42 articles after obtaining the full text. Finally, we selected 32 articles by personal searching and use of references. Thus, we assessed a total of 819 articles (Figure 1).

### Characteristics of the Included Articles

Table 1 details the characteristics of the included articles. The selected articles assessed mainly treatments in the field of cardiology (123/819 [15%]), endocrinology, nutrition and dietetics (88/819 [11%]); musculoskeletal system (80/819 [10%]); anesthesiology, pain, and critical care (78/819 [9%]); and the respiratory system (63/819 [8%]). The experimental treatments concerned mainly oral drugs and topical treatments (508/819 [62%]) and injections such as intravenous, intramuscular, or intra-articular treatment (178/819 [22%]). A total of 613 reports (75%) described a placebo control treatment in at least one arm of the trial, and 196 (32% of the placebo-controlled trials) the content of the placebo (e.g., sugar, saline solution).

**Table 1:**
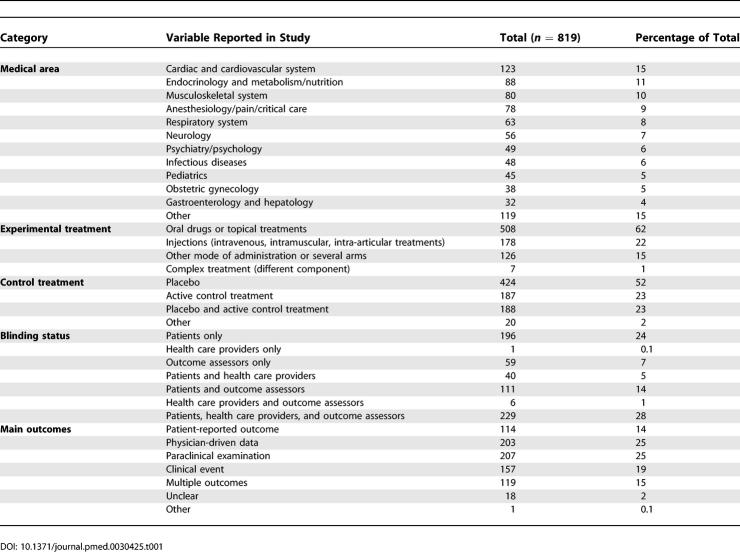
Characteristics of the Articles Selected for Assessing Blinding in RCTs

The main outcomes reported were patient-reported outcomes (114/819 [14%]), physician-driven data (203/819 [25%]), complementary investigations (207/819 [25%]), clinical events (157/819 [19%]), and multiple outcomes (119/819 [15%]). The main outcome was unclear in 18 articles (2%).

A total of 576 reports (71%) clearly reported blinding for patients, 276 (34%) health care providers, and 405 (50%) outcome assessors. Success of blinding was described in 35 reports (5%).

### Method of Blinding

More than half of the reports (472 [58%]) described the method of blinding, but 236 (29%) gave no detail and 111 (13%) some data on blinding (i.e., reporting that treatments were similar or the use of double dummies with no description of the method). The methods of blinding identified varied in complexity.

### Methods to Establish Blinding of Patients or Health Care Providers

**Figure 2:**
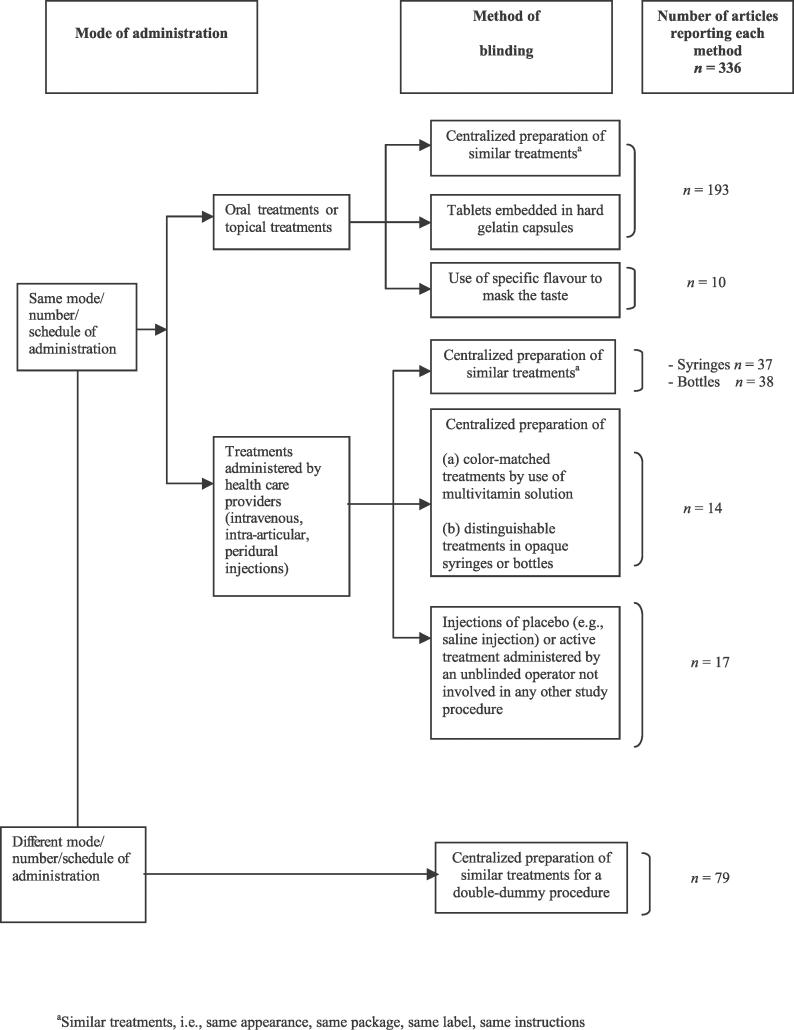
Methods to Establish Blinding in RCTs Methods of blinding of patients or health care providers (i.e., to provide indistinguishable treatments) were determined for published reports of RCTs of pharmacologic treatment.

A total of 336 reports (41%) described methods to establish blinding of patients or health care providers (Figure 2). The authors reported use of a centralized preparation of similar capsules, tablets, or embedded treatments in hard gelatin capsules (193/336 [57%]), similar syringes (37/336 [11%]), or similar bottles (38/336 [11%]). Use of a double dummy procedure was described in 79 articles (23%). Other methods consisted of a sham intervention performed by an unblinded health care provider who was not actively involved in the care of patients and had no other contact with patients or other caregivers and outcome assessors (17/336 [5%]). To mask the specific taste of the active treatments, in ten articles researchers used a specific flavor such as peppermint or sugar to coat treatments. For treatments administered by care providers, authors reported use of a centralized preparation of opaque coverage to adequately conceal intravenous treatments with different appearances (14/336 [4%]). Appendix 1 in Protocol S1 gives examples of these methods.

### Methods to Avoid Unblinding of Patients or Health Care Providers

**Figure 3:**
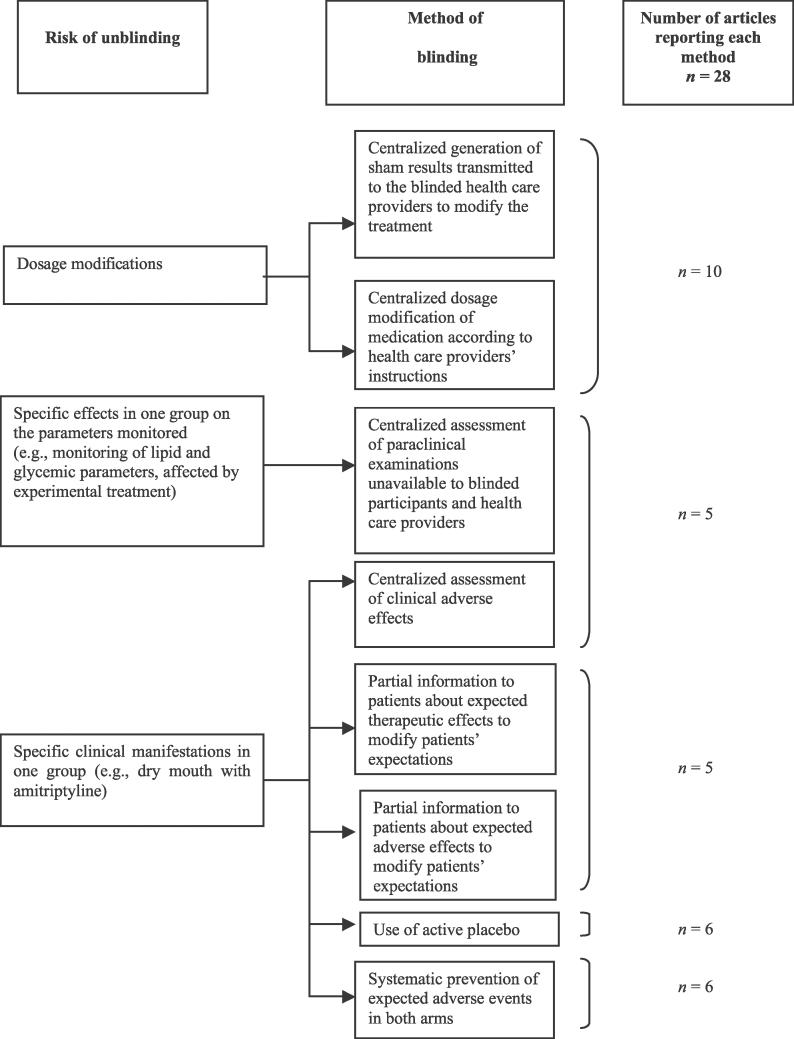
Methods to Avoid Unblinding Methods to avoid unblinding of patients or health care providers (i.e., to avoid unblinding during the trials because of specific side effects or specific requirements such as dosage modification) were determined in published reports of RCTs of pharmacologic treatment.

Very few studies overall (28/819 [3%]) reported methods to avoid unblinding. Figure 3 presents these methods, which concern only treatments necessitating dosage adaptation or those with frequent and/or specific side effects. These methods relied on use of a centralized adapted dosage or provision of sham results of complementary investigations for treatments necessitating dosage adaptation.

Methods to avoid unblinding because of side effects relied mainly on centralized assessment of side effects, partial information to patients about side effects, use of active placebo or systematic prevention of adverse effects in both arms. Appendix 2 in Protocol S1 gives examples of methods to avoid unblinding of patients or health care providers.

### Methods of Blinded Assessment

**Figure 4:**
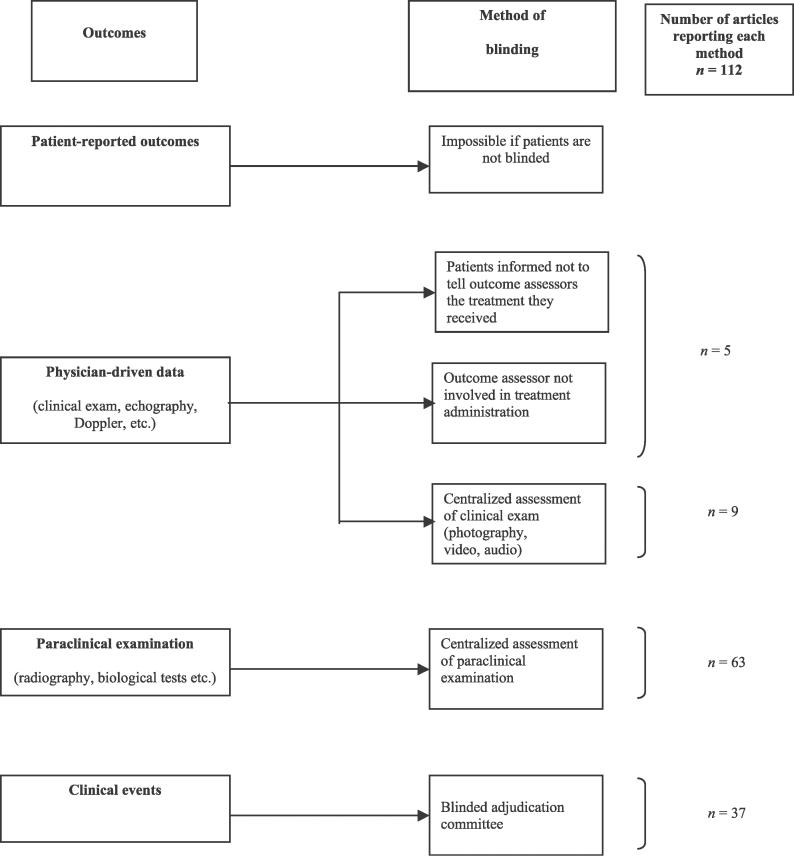
Methods of Blinded Outcome Assessment in Published Reports of RCTs Assessing Pharmacologic Treatment

These methods depend on the main outcomes and are particularly useful when blinding cannot be established and maintained by the methods described previously. A total of 112 articles (14%) described these methods, which relied mainly on a centralized assessment of the main outcome (Figure 4). Appendix 3 in Protocol S1 gives examples of these methods. Blinding of outcome assessors will presumably be achieved if neither patients nor those involved in the trial have any means to discover which arm a patient is in, for example because the placebo and active drugs are indistinguishable and allocation is via a central randomization service. Specific measures to blind outcome assessors may thus be required mainly when the nature of the trial is such that complete blinding during the earlier stages is not feasible. However, in this sample, 96 reports (86%) over the 112 reports in which specific measures to blind the outcome assessor were reported concern trials in which patients were reported as blinded or in which double blinding or triple blinding was reported. These results suppose that although blinding was performed at an earlier stage, trialists nevertheless decided to perform a specific method of blinding the outcome assessor.

## Discussion

This study assessed the reporting of the methods of blinding in RCTs of pharmacologic treatment and provides a description and classification of methods of blinding patients, health care providers, and outcome assessors based on a large cohort of RCTs. We identified 819 reports with about 60% describing the method of blinding. Our classification identified three main methods of blinding: (1) methods to provide identical treatments in both arm, (2) methods to avoid unblinding during the trial, and (3) methods of blinded outcome assessment.

Although blinding is essential to avoid bias, the reporting of blinding is generally quite poor [5] and reviews of trials that test the success of blinding methods indicate that a high proportion of trials are unblinded [14,15]. Lack of reporting of blinding [5,14–16] is probably linked in part to the lack of awareness of existing methods of blinding. To improve the performance and reporting of the method of blinding, we established an inventory of blinding methods of patients, health care providers, and main outcome assessors and classified them mainly on the basis of what seemed reasonable after having read through the various methods. Although other ways of classifying could be considered, we decided to focus on the mode of administration of the treatments, the risk of unblinding, and the primary outcomes, as these are more practical and logical criteria for researchers when planning trials.

Our classification emphasizes that blinding outcome assessors depends mainly on the primary outcome. In most situations, blinding outcome assessors should therefore be possible with a centralized assessment of complementary investigation, physician-mediated data, and clinical events (Figure 4). For example, for the assessment of gastroscopy, endoscopic procedures could be completely recorded using a VHS recorder and evaluated under blinded conditions [17]. In a trial comparing the use of two analgesic treatments during a vaccination procedure of children, the procedure was videotaped. A mirror was mounted on the wall behind the examining table so that the observer could film the infant's reaction both face on and from the mirror image. A trained observer, who was unaware of the treatment assignment, scored the pain of vaccination from the videotapes using the Modified Behavioral Pain Scale [18]. These methods are probably more successful than the method of informing patients not to tell outcome assessors the treatment they received. In fact, a study comparing conservative treatment (splinting) with surgery in the treatment of carpal tunnel syndrome involved an attempt to blind the outcome assessor by encouraging the patients not to reveal their treatment and by masking the surgical scar. Despite these efforts, the assessor correctly guessed the treatment performed [19]. Finally, for patient-reported outcomes for which the patient is the outcome assessor, if no methods can overcome the difficulties in establishing and maintaining blinding, no methods should be used so as to avoid ascertainment bias by a blinded outcome assessment.

This systematic review also highlights some creative methods of blinding that are frequently used in specific areas and could overcome some barriers to blinding. For example, in a trial comparing ximelagatran and warfarin for the prevention of venous thromboembolism, to avoid unblinding an anticoagulation management center relayed real or sham values to health care providers [20]. In a trial comparing the efficacy of infliximab versus placebo for psoriatic arthritis, to offer active medication to patients randomized to receive placebo, patients with less than 10% improvement from baseline entered early escape and received infliximab at weeks 16, 18, and 22. To maintain blinding, patients randomized to receive infliximab who had less than 10% improvement received additional placebo infusions at weeks 16, 18, and 22 [21]. Some reports have proposed use of active placebo. Active placebos are designed and used to mimic some of the side effects of the intervention to further secure blinding [22–26]. For example, in a trial to investigate the immunologic response of three doses of cat hair compared to placebo, researchers added caramelized sugar and histamine to the placebo and to the two lowest doses of cat extract to simulate the local reaction of the most concentrated extract [27].

However, some of these methods might be debatable from an ethical point of view. For example, use of active placebo is ethically debatable, and researchers should consider the balance between the expected harm linked to the placebo and the expected benefit of administering an active placebo [26]. Further, some ethics committees might reject a study involving blinding patients to some hypotheses such as not informing them about side effects of medication. However, this method might be acceptable if patients are informed about all the possible side effects but not informed of specific side effects of the treatment administered.

The classification and dissemination of these methods to researchers could overcome some barriers of blinding. Consequently, this classification (Figures 2–4) and the detailed examples of reporting (Protocol S1) should be helpful to researchers in planning trials as well as to readers and reviewers in appraising the feasibility of blinding in published reports of clinical trials.

Finally, our results also concludes that only 58% of the reports provided some details of the methods used to blind patients, health care providers, and outcome assessors. These results are consistent with those of Ferguson et al. [16], who showed that matching of the characteristics of placebo to the intervention was reported in 53% of trials. These results might be explained in part by the insufficient coverage of blinding in the Consolidated Standards for Reporting Trials (CONSORT) statements. In fact, empirical evidence has demonstrated that lack of blinding and allocation concealment could bias the results [4,6,28]. To take into account these issues, three items of the CONSORT statements are dedicated to the description of the randomization procedure, whereas only one item is dedicated to the blinding issue [29]. The CONSORT statements mainly focus on reporting who is blinded [2] and less on the reporting of details on the method of blinding, whereas this information is essential to appraise the success of blinding. In fact, some evidence suggests that although participants are reported as blinded, the success of blinding might be questionable [11]. For instance, in a study assessing zinc treatment for the common cold, the blinding procedure failed, because the taste and aftertaste of zinc was distinctive [11,30]. Nevertheless tools used to assess the quality of trials included in meta-analyses and systematic reviews mainly focus on the reporting of the blinding status for each participant [31–33], and rarely on the description of the blinding methods and the adequacy of the blinding method [34]. Therefore, we must strengthen the reporting guidelines related to blinding issues, emphasizing adequate reporting of the method of blinding.

In this study, we assessed reports of RCTs published in high impact-factor journals from 2004. Consequently, we focused only on a specific panel of RCTs and on the reporting of these trials, not the trials themselves [29,35]. For example, we could not provide data on the methods of blinding data collectors, because these data were poorly reported, and we were unable to consistently identify data collectors in the selected articles. In addition, if the method of blinding was detailed in another article, we did not check for the description of the method. Thus, some methods of blinding might have been missed. However, the high number of contemporary trials included implies that we described a substantial number of blinding methods and also highlighted creative methods of blinding. Furthermore, these results are evolving, and readers are invited to inform us of other methods of blinding not captured in this survey. Finally, we did not focus on other key trial participants, such as data analysts. However, there is no reason that data analysts could not be blinded, and therefore the identification and dissemination of specific methods of blinding data analysts is probably not necessary.

In conclusion, this study highlights two important issues. First, our detailed description and classification of several ways to establish and maintain blinding helps ensure a blinded outcome assessment in RCTs. These methods should be disseminated to researchers to overcome some of the barriers to blinding in clinical trials. Second, we confirmed the insufficient reporting of the methods of blinding and the need to increase the requirement related to blinding issues in the CONSORT statements.

## Supporting Information

Protocol S1 Appendices Three appendices are provided. Appendix 1 reports examples of the methods used to establish blinding in the selected published reports of randomized controlled trials. Appendix 2 focuses on the examples of the methods to avoid unblinding in randomized controlled trials. Appendix 3 provides examples of the methods to blind outcome assessors.
(140 KB DOC)

## Abbreviations

CONSORT: Consolidated Standards for Reporting Trials
RCT: randomized controlled trial
