# Supplementary material for: Methods of Blinding in Reports of Randomized Controlled Trials Assessing Pharmacologic Treatments: A Systematic Review
Source: PLoS Med. 2006 Oct 31;3(10):e425. doi: 10.1371/journal.pmed.0030425 (PMC1626553; doi:10.1371/journal.pmed.0030425)
Supplement: Protocol S1 — Three appendices are provided. Appendix 1 reports examples of the methods used to establish blinding in the selected published reports of randomized controlled trials. Appendix 2 focuses on the examples of the methods to avoid unblinding in randomized controlled trials. Appendix 3 provides examples of the methods to blind outcome assessors. (140 KB DOC) [file pmed.0030425.sd001.doc]

Protocol S1

Appendix 1: Methods to establish blinding in published reports of randomized controlled trials of pharmaceutical treatments

|  | Examples |
| --- | --- |
| - Centralized preparation of similar treatments such as similar treatments or tablets embedded in hard gelatin capsules | 1) Because it is infeasible to obtain placebo tablets identical to Zocor tablets, the Central Pharmacy manufactured placebo tablets for the study, and both Zocor and the placebo tablets were embedded in a hard gelatin capsule with a similar appearance to the active medicine and placebo.^1^  2) Placebo tablets were provided by Merck and were matched in size and shape. All study subjects were given identical dosage instructions. ^2^  3) Central teams pre-packed active and placebo treatments in monthly doses and stored all four packs for each patient in a sealed numbered envelope. The placebo tablets were manufactured to be the same size, shape, and color as the active tablet.^3^  4) Active treatments were provided by the respective companies. Boxes containing medication for periods between the visits were prepared by employees of the hospital pharmacy not involved in the design and performance of the study. Tablets were crushed and the powder was placed in easy-to-swallow water containers. Lactose powder was used as a placebo. Each box contained two similar bags containing an appropriate number of containers. Bags were not labeled and boxes were labeled with the patient number only. Attribution of each box to a treatment group was known solely to the hospital pharmacy employees involved in their preparation.^4^ |
|  |  |
| - Centralized preparation of similar treatments such as similar topical application | 1) In an RCT comparing fluorouracil cream and placebo, the active and placebo creams were indistinguishable in texture, color, and smell and were supplied in identical tubes, patient kits and labeling. |
|  |  |
| - Use of specific flavor to mask the taste | 1) use of wild-cherry-flavored syrup^5^  2) use of peppermint^6^  3) Sugar coated^7, 8^  4) In a trial comparing zinc and placebo, the placebo syrup consisted of the syrup base used for the zinc mixture, which was appropriately modified by use of edible fruit (myrobalan) with an astringent taste to give similar taste to the zinc mixture.^9^  5) other examples^10-13^ |
| - Centralized preparation of distinguishable treatments in opaque syringes or bottles | 1) In a trial comparing intravenous iron dextran therapy for restless legs syndrome versus placebo, blinding was obtain by use of infusion pump with the medication (or placebo) and tubing covered with an opaque obscuring sleeve so that the patient, investigator and study nurse could not detect which was being administered.^14^  2) Opaque covers were used to conceal the infusion bags and catheters used for infusion. The placebo infusions were color-matched to the corresponding treatment by use of a multivitamin solution to create a yellow solution^15^.  3) Immunoglobulin and placebo infusion ’bags were prepared by the unblinded pharmacist and delivered to the clinical unit in bottles with special plastic covers. A small vertical slit allowed the nurse to watch the level of infusion without seeing the bubbles of the immunoglobulin infusion.^16^ |
| - Injections of placebo or active treatments administered by an unblinded operator not involved in any other study procedure | 1) The injection (methylprednisolone or saline solution) was constituted and administered by a nurse who was not blinded to the treatment but who had no involvement in any aspect of the study. This individual was instructed not to provide the patients, physician or study personnel with any information about the contents of the syringe. The nurse was also instructed not to allow anyone to see the content of the syringe. The injections were administered in a private setting with no one else present.^17^  2) Since the vaccines compared are distinct in appearance and volume, special precautions were taken to ensure the double-blind nature of the trial. A vaccination team prepared the vaccine and masked the content of the syringe with opaque tape before immunization. This team was not involved in any other study procedures, including surveillance for endpoints.^18^  3) In a trial comparing neurolytic celiac plexus block and systemic analgesic therapy alone, blinding of patients was obtained by use of a sham procedure involving subcutaneous and intramuscular 0.5% bupivacaine injection at a typical NCPB site. This procedure was performed with the identical room and set-up, prone positioning, fluoroscopy machine movement, personnel and timing as the actual NCPB. Sham images appeared on the computer screen, but no actual fluoroscopy was used. The study team consisted of members with unique roles to maintain the study blinding: the clinical manager was a physician blinded to the randomized intervention who was responsible for all pain management decisions including dosage of analgesic medications. The observer was a clinical nurse blinded to the randomized intervention who performed all patient assessments. The operator was a physician not blinded to the randomized intervention who performed the NCPB or sham procedure. Following the randomized procedure, the operator was not actively involved in the care of that patient. Other medication decisions were made by the patients’ primary physicians who were blinded to treatment assignment.^19^  4) In a trial assessing intravitreal injection into one eye or sham injection administered every 6 weeks over 48 weeks, to maintain masking, the patients receiving sham injections and those receiving the active treatment were treated identically, with the exception of scleral penetration. All patients (including those receiving sham injection) underwent an ocular antiseptis procedure and received injected subconjunctival anesthetic. The patients receiving sham injections had an identical syringe -- but without the needle -- pressed against the eye wall to mimic the active doses that were injected through the pars plana into the vitreous cavity. The injection technique precluded the patient from seeing the syringe. To maintain blinding of the investigators, the study ophtalmologist responsible for patient care and for the assessment did not administer the injection^20^  5) Other examples^21-31^ |
|  |  |
| - use of double dummy | 1) In a trials comparing the efficacy of sublingual birch pollen allergen-specific immunotherapy (SLIT group) and subcutaneous birch pollen allergen-specific immunotherapy (SCIT group), patients were allocated into 3 arms^32^:   - SLIT arm receiving subcutaneous placebo and immunotherapy sublingual drops; - SCIT group receiving subcutaneous immunotherapy and placebo sublingual drops; - the placebo arm receiving subcutaneous placebo and placebo sublingual drops.   2) In a trial assessing the efficacy of abciximab infusion^33^ for 24 hours or 48 hours versus placebo, patients were allocated into groups with the following treatments:   - abciximab infusion for 24 hours followed by 24 hours of placebo infusion - abciximab infusion for 48 hours - placebo infusion for 48 hours   3) In a trial assessing the efficacy of etoricoxib, 120 mg, once daily compared to indomethacin, 50 mg, three times daily, patients were allocated into groups with the following treatments^34^:   - placebo indomethacin (3 doses) and placebo etoricoxib (1 dose) - active indomethacin (3 doses) and placebo etoricoxib (1 dose) - placebo indomethacin (3 doses) and active etoricoxib (1 dose) |
| - Imperfect blinding method | 1) Treatments differ in appearance and smell. Active gel is brownish in color and has characteristic odor. Because making both treatments identical or using the double dummy technique is intrinsically impossible, the original tube of active and control treatments were used. Masking was achieved by sealing the tubes in a double layer of opaque blue plastic. The standard blue-ribbed caps of the control tubes were replaced by white caps of a different shape and size. The patients received a sealed numbered box containing the study medication and rescue analgesics and were instructed to open it only on returning home, thus ensuring that the clinical metrologist did not see its content.^35^ |

Appendix 2: Methods to avoid unblinding in randomized controlled trials of pharmaceutical treatments

|  | Examples |
| --- | --- |
| - Dosage modification |  |
| - Centralized generation of sham results | 1) In a trial comparing ximelagatran and warfarin for the prevention of venous thromboembolism, to maintain blinding, INR values were measured either at the participating centers with the use of encrypted point of care devices or at the local laboratories equipped with a system that prevented access to the INR value by local personnel. All INR values were reported to an anticoagulation management center, which relayed real or sham values to the local personnel. For patients receiving ximelagatran, sham values were generated to mimic the usual values in patients receiving warfarin.^36^  2) In a trial comparing low-intensity warfarin therapy with conventional-intensity warfarin therapy for long-term prevention of recurrent venous thromboembolism, all INR measurements were forwarded only to the center-specific anticoagulation monitor. For patients assigned to conventional-intensity warfarin therapy, the true INR was relayed to the clinical center. For those assigned to the low-intensity warfarin therapy, the anticoagulation monitor converted the INR result to a higher value and relayed this converted result to the clinical center. This procedure allowed patients to receive warfarin adjusted to achieve an INR of 1.5-1.9 or 2.0-3.0 while maintaining the blinding of patients and care providers.^37^  3) In a trial comparing a target functional oxygen-saturation range, either 91-94% (low-saturation group) or 95-98% (high-saturation group) for extremely preterm infants. To ensure blinding, the infants who underwent randomization were assigned a specific study oximeter, which after calculation of the infant’s oxygen saturation level in the usual manner, was adjusted to display a value 2 percent higher than the actual saturation in the high-saturation group. Staff members and parents were asked to target the range between 93% and 96% for the infant’s saturation so that they remained unaware of treatment assignment. Care givers were aware that they were using adjusted oxymeters and that they were participating in a trial, but they were not aware of the offset level of the individual oxymeter.^38^  4) In a trial comparing the efficacy of infliximab versus placebo for psoriatic arthritis, to offer active medication to patients randomized to placebo, any patients with less than 10% improvement above baseline entered an early escape phase and received infliximab at weeks 16, 18, 22. To maintain blinding, patients randomized to receive infliximab who had less than the 10% improvement received additional placebo infusion at weeks 16, 18, 22.^39^  5) other examples:^40-43^ ^44-46^ |
| - Centralized dosage modification of medication | 1) Double-blind placebo-controlled trials performed to evaluate the efficacy of amitriptyline. An active placebo based on its ability to produce dry mouth, a common side effect of amitriptyline, was used (benztropine)^47^. All patients were telephoned by the research nurse to assess pain and medication side effects. The nurse then communicated with the pharmacy as if each patient were under active treatment. If patients reported bothersome side effects, the nurse asked the pharmacy to decrease the dosage to the previous dosage level; if side effects were minimal or tolerable, the pharmacy was instructed to increase the dosage according to the protocol. If the medication was the active placebo, the pharmacy made no change. The pharmacy did not inform the research nurse of the patients’ medication or the change made. |
| - Methods to mask side effects |  |
| - centralized assessment of side effects | 1) In a trial comparing estrogen plus progestin compared to placebo, to prevent unblinding of clinical staff, breast discomfort and vaginal bleeding were reported directly to gynecological staff who were located separate from the clinical center, did not communicate with the clinical center personnel about gynecological symptoms, and did not participate in ascertainment of cardiovascular outcomes.^48^  2) Patients were randomized to receive 2 different active treatments (cannabis extract, tetrahydrocannabinol) or a placebo for the treatment of spasticity related to multiple sclerosis. To detect any illicit cannabis use, urine samples were collected and assayed for cannabinoid. To maintain blinding, at the coordinating center, all blood and urine results were held at an independent site until statistical analysis began.^49^  3) Doxycycline levels were determined to help assess adherence to doxycycline therapy. Coded blood samples were transferred from the central laboratory to the doxycycline testing laboratory.^50^  4) To ensure masking, lipid and glycaemic parameters, which could have been affected by experimental treatment, were measured at a central laboratory and were unavailable to participants and doctors for the duration of the trial.^51^  5) To assess the effect of simvastatin vs placebo on bone turnover and BMD, the investigator responsible for the clinic visits was unaware of the results of laboratory measurements (plasma cholesterol level).^52^  6) Other^44^ |
| - Partial information to patients about expected therapeutic effects - Partial information to patients about expected adverse effects | 1) double-blind placebo-controlled trials comparing 2 doses of treatment and a placebo. Patients were informed about the study procedure and that they would be given, in a blinded fashion, a dose of the study medication. They were informed about potential side effects but not about any potential actions of the medication.^53^  2) The label appearance was identical, but the flavor was different. Patients were not informed about the taste of the active treatment and the placebo.^54^  3 In a trial comparing intravitreal injection of triamcinolone acetonide to placebo for persistent diabetic macular edema after adequate laser treatment, to mask patients, they were advised that floaters, which are prominent after an injection of triamcinolone, may occur after an injection of placebo as well as the active study medication.^55^  4 patients were given the expectation that they would receive capsules of fish oil (either concentrated or deodorized and concentrated) or olive oil (pure or with fishy flavoring added to it). In reality, subjects received either concentrated fish oil or pure olive oil.^56^ |
| - use of active placebo | 1) Double-blind placebo-controlled trials performed to evaluate the efficacy of amitriptyline. An active placebo based on its ability to produce dry mouth, a common side effect of amitriptyline, was used (benztropine).^47^  2) In a trial performed to investigate immunologic responses with 3 serial doses of cat hair, caramelized sugar and histamine were added to the placebo and the 2 lowest doses of cat extract so that they simulated the most concentrated extract in both color and tendency to produce a local reaction.^57^  3) Other examples^32, 58-60^ |
| - other | 1) Because doxycycline can cause photosensibility to sunlight, all participants were provided with sunscreen and were advised to limit sun exposure. ^50^  2) To prevent potential unblinding due to nausea and vomiting associated with the use of cyclophosphamide, it was recommended that all patients be administered antiemetic agent one hour before the infusions of cyclophosphamide or the placebo of cyclophosphamide.^61^  3) In a trial comparing prednisolone to placebo for the treatment of adhesive capsulitis, patients with diabetes mellitus were excluded because of the potential for unblinding.^62^  4) The crossover trial comparing use of theophylline to placebo for sleep adopted an unusual design to minimize the danger of side effects destroying blinding. Side effects (nausea, headaches, nervousness) usually occur during the first few days with theophylline therapy and then wear off. Patients were therefore treated with theophylline for a few days before passing onto each arm of the trial. Patients remaining under theophylline would already be used to it and those under placebo would be unaware of the switch. The crossover study had an extra non-blind phase in between the usual 2 10-day blind phases, in which all patients took their optimal dose of theophylline for 7 days. Thus, no patients began theophylline de novo during a blind phase.^63^ |

Appendix 3: Method to blind outcome assessors in randomized controlled trials of pharmaceutical treatments

|  | Examples |
| --- | --- |
| - - patients informed not to tell outcome assessor the treatment they received | 1) Differences in viscosity of the solution or specific patients’ reaction to the treatment were likely to reveal treatment assignment. “patients and unmasked personnel were cautioned not to reveal the treatment assignment or frequency of administration to the masked evaluator. The study records indicating treatment regimen assignment were not available to masked personnel.”^64^  2) Patients were assigned to receive intensive or routine management. Before every assessment, patients were sent a letter stating that they should make no mention of their drug treatment or the identity of their doctor. Assessments were undertaken without the case record and on a different clinic day from that attended by the intensive group for their monthly visit. The assessing metrologist did not attend the other outpatient clinics, at which the intensive group attended every month, and did not take part in intensive treatment.^65^  3) Because of the milky appearance of the prednisolone, we faced some barriers to guarantee the masked fashion of this study. To overcome this problem, the observers were unaware of any details of the patient treatment and the patients were asked not to provide any information regarding eyedrop instillation.^66^  4) Other examples ^44, 54, 67-69^ |
| - - Outcome assessor not involved in treatment administration | 1) The investigator obtaining patient data (pulmonary function, exercise capacity etc.) was not present during treatment administration (i.e., administration of a different volume of lactate ringer solution).^21^  2) Physicians responsible for weaning patients from the ventilator were absent at the time of the study-drug administration.^70^  3) Because of a potential for loss of patient blinding with active medication, a treating doctor and assessor had to be present at each center. The treating doctor monitored the dose, side effects and patients’ well-being, and the assessor assessed the main outcomes: Ashworth score of spasticity.^49^ |
| - - centralized assessment of clinical exam | 1) The vaccination procedure was videotaped with a color camera. A mirror was mounted on the wall behind the examining table so that the observer could film the infant’s reaction both face on and from the mirror image. The observer stood about 3 ft from the infant and did not interfere with the procedure. The entire vaccination procedure was taped until the infant settled down. A trained observer, who was unaware of the treatment assignment, scored the pain of vaccination from the videotapes on the Modified Behavioral Pain Scale.^71^  2) The anaesthesia induction period until 10 min after placement of tracheal tube was videotaped and reviewed by a blinded independent paediatric neurologist to assess the presence of agitation.^72^  3) Patients’ responses to clozapine and risperidone were videotaped for subsequent coding by blinded raters on 5 specific dimensions of verbal behavior (clarity, focus, negotiation, persistence and overall conversation), and 2 nonverbal dimensions (fluency, affect).^73^  4) The main outcome was the glabellar lines evaluated by an independent committee of experts by examination of standardized photographs at rest taken from each patient before and one month after treatment, the experts being blinded to the treatment and to the time of the photographs.^74^  5) Cutaneous reactions were photographed and reviewed by the scientific committee.^75^  6) The main outcome was change from baseline in iris color and eyelash length and density. The iris and eyelid was photographed for each patient. A standardized camera system was provided to each investigator, and the study had a standardized procedure to place the patient for the photography. Photographic slides were then forwarded to an independent central reading facility for interpretation in a masked fashion. A photographic compact disk was also archieved.^76^ |
| - - centralized assessment of paraclinical examination (main outcome or part of the main composite outcome) | 1) Laboratory evaluations were performed at a central laboratory.^77^  2) Liver biopsy was performed at transplantation and after 12 months. Slides were made available for a central reading by an assessor blinded to the trial treatment.^78^  3) The structure of the osteoarthritic knee was evaluated by radiography. All films were analyzed centrally by two blinded observers.^22^  4) MRI analysis was performed at a single center blinded to treatment assignment.^79^  5) BMD scans of the hip and lumbar spine were performed. Digitalized data were analyzed, without knowledge of treatment assignment, at a central reading center.^80^  6) All ECG angiograms were submitted to an independent ECG core laboratory blinded to the treatment assignment and to the angiographic core laboratory where they were analysed with use of a computer-based system.^81^  7) Echocardiograms were videotaped and sent for central analysis at a core laboratory. Analyses were done off-line on a commercial digitizing system, that was blinded to treatment allocation and clinical characteristics.^82^  8) Arthroscopy was recorded on a videocassette. All the videotapes were analyzed by a single blinded investigator.^83^  9) Each endoscopic procedure was completely recorded using a VHS recorder and evaluated by an independent gastroenterologist and a medical advisor, for gastroscopy under blind conditions.^84^  10) The primary outcome was the change from baseline in mean carotid intima-medial thickness. One experienced blinded sonographer performed all B-mode ultrasonography. The sonographer saved a video still image of each segment as a 4:1 compressed JPEG file. At the end of the study, 1 image-blinded analyst batch-read all ultrasound images.^85^  11) Other complementary tests: radionuclide ventriculography^86^, mammography^87^ |
| - - Blinded adjudication committee | 1) “The primary efficacy variable was the proportion of patients developing CMV disease during the first six months post-transplantation as adjudicated by an independent blinded endpoint committee (medically qualified individuals with expertise in CMV infection in transplant recipients).”^88^  2) All thrombotic events were adjudicated by a blinded central adjudication committee of two experts.”^41^  3) The intravascular ultrasonography was performed after the interventional procedure and at follow-up. Continuous imaging was performed as the intravenous ultrasonography (IVUS) catheter was automatically withdrawn at 0.5 mm/s by use of a motorized pullback device. The images were recorded on a VHS videotape for off-line analysis. IVUS measurements were performed by an independent core laboratory blinded to treatment assignment.^89^  4) A clinical evaluation committee, using prospectively defined criteria, evaluated the case report form of each patient in a blinded fashion^90^ |

References

**1.** Rejnmark L, Buus NH, Vestergaard P, et al. Effects of simvastatin on bone turnover and BMD: a 1-year randomized controlled trial in postmenopausal osteopenic women. *J Bone Miner Res.* 2004;19:737-744.

**2.** Wenstrup RJ, Bailey L, Grabowski GA, et al. Gaucher disease: alendronate disodium improves bone mineral density in adults receiving enzyme therapy. *Blood.* 2004;104:1253-1257.

**3.** Sankar V, Brennan MT, Kok MR, et al. Etanercept in Sjogren's syndrome: a twelve-week randomized, double-blind, placebo-controlled pilot clinical trial. *Arthritis Rheum.* 2004;50:2240-2245.

**4.** Kurowski M, Kuna P, Gorski P. Montelukast plus cetirizine in the prophylactic treatment of seasonal allergic rhinitis: influence on clinical symptoms and nasal allergic inflammation. *Allergy.* 2004;59:280-288.

**5.** Bjornson CL, Klassen TP, Williamson J, et al. A randomized trial of a single dose of oral dexamethasone for mild croup. *N Engl J Med.* 2004;351:1306-1313.

**6.** Theobald HE, Chowienczyk PJ, Whittall R, Humphries SE, Sanders TA. LDL cholesterol-raising effect of low-dose docosahexaenoic acid in middle-aged men and women. *Am J Clin Nutr.* 2004;79:558-563.

**7.** Muller T, Mannel M, Murck H, Rahlfs VW. Treatment of somatoform disorders with St. John's wort: a randomized, double-blind and placebo-controlled trial. *Psychosom Med.* 2004;66:538-547.

**8.** Muller-Fassbender H, Bach GL, Haase W, Rovati LC, Setnikar I. Glucosamine sulfate compared to ibuprofen in osteoarthritis of the knee. *Osteoarthritis Cartilage.* 1994;2:61-69.

**9.** Mahalanabis D, Lahiri M, Paul D, et al. Randomized, double-blind, placebo-controlled clinical trial of the efficacy of treatment with zinc or vitamin A in infants and young children with severe acute lower respiratory infection. *Am J Clin Nutr.* 2004;79:430-436.

**10.** Argyropoulos SV, Hood SD, Adrover M, et al. Tryptophan depletion reverses the therapeutic effect of selective serotonin reuptake inhibitors in social anxiety disorder. *Biol Psychiatry.* 2004;56:503-509.

**11.** Anderson TW, Reid DB, Beaton GH. Vitamin C and the common cold: a double-blind trial. *Can Med Assoc J.* 1972;107:503-508.

**12.** Prasad AS, Fitzgerald JT, Bao B, Beck FW, Chandrasekar PH. Duration of symptoms and plasma cytokine levels in patients with the common cold treated with zinc acetate. A randomized, double-blind, placebo-controlled trial. *Ann Intern Med.* 2000;133:245-252.

**13.** Rasmussen MH, Andersen T, Breum L, Gotzsche PC, Hilsted J. Cimetidine suspension as adjuvant to energy restricted diet in treating obesity. *Bmj.* 1993;306:1093-1096.

**14.** Sloand JA, Shelly MA, Feigin A, Bernstein P, Monk RD. A double-blind, placebo-controlled trial of intravenous iron dextran therapy in patients with ESRD and restless legs syndrome. *Am J Kidney Dis.* 2004;43:663-670.

**15.** Walsh TS, McArdle F, McLellan SA, et al. Does the storage time of transfused red blood cells influence regional or global indexes of tissue oxygenation in anemic critically ill patients? *Crit Care Med.* 2004;32:364-371.

**16.** Cordonnier C, Chevret S, Legrand M, et al. Should immunoglobulin therapy be used in allogeneic stem-cell transplantation? A randomized, double-blind, dose effect, placebo-controlled, multicenter trial. *Ann Intern Med.* 2003;139:8-18.

**17.** Lahn M, Bijur P, Gallagher EJ. Randomized clinical trial of intramuscular vs oral methylprednisolone in the treatment of asthma exacerbations following discharge from an emergency department. *Chest.* 2004;126:362-368.

**18.** Alonso PL, Sacarlal J, Aponte JJ, et al. Efficacy of the RTS,S/AS02A vaccine against Plasmodium falciparum infection and disease in young African children: randomised controlled trial. *Lancet.* 2004;364:1411-1420.

**19.** Wong GY, Schroeder DR, Carns PE, et al. Effect of neurolytic celiac plexus block on pain relief, quality of life, and survival in patients with unresectable pancreatic cancer: a randomized controlled trial. *Jama.* 2004;291:1092-1099.

**20.** Gragoudas ES, Adamis AP, Cunningham ET, Jr., Feinsod M, Guyer DR. Pegaptanib for neovascular age-related macular degeneration. *N Engl J Med.* 2004;351:2805-2816.

**21.** Holte K, Klarskov B, Christensen DS, et al. Liberal versus restrictive fluid administration to improve recovery after laparoscopic cholecystectomy: a randomized, double-blind study. *Ann Surg.* 2004;240:892-899.

**22.** Pham T, Le Henanff A, Ravaud P, Dieppe P, Paolozzi L, Dougados M. Evaluation of the symptomatic and structural efficacy of a new hyaluronic acid compound, NRD101, in comparison with diacerein and placebo in a 1 year randomised controlled study in symptomatic knee osteoarthritis. *Ann Rheum Dis.* 2004;63:1611-1617.

**23.** Angst MS, Ramaswamy B, Davies MF, Maze M. Comparative analgesic and mental effects of increasing plasma concentrations of dexmedetomidine and alfentanil in humans. *Anesthesiology.* 2004;101:744-752.

**24.** Auer J, Weber T, Berent R, et al. A comparison between oral antiarrhythmic drugs in the prevention of atrial fibrillation after cardiac surgery: the pilot study of prevention of postoperative atrial fibrillation (SPPAF), a randomized, placebo-controlled trial. *Am Heart J.* 2004;147:636-643.

**25.** Kalunian KC, Moreland LW, Klashman DJ, et al. Visually-guided irrigation in patients with early knee osteoarthritis: a multicenter randomized, controlled trial. *Osteoarthritis Cartilage.* 2000;8:412-418.

**26.** Hasso N, Maddison PJ, Breslin A. Intra-articular methotrexate in knee synovitis. *Rheumatology (Oxford).* 2004;43:779-782.

**27.** Altman RD, Akermark C, Beaulieu AD, Schnitzer T. Efficacy and safety of a single intra-articular injection of non-animal stabilized hyaluronic acid (NASHA) in patients with osteoarthritis of the knee. *Osteoarthritis Cartilage.* 2004;12:642-649.

**28.** Apfel CC, Korttila K, Abdalla M, et al. A factorial trial of six interventions for the prevention of postoperative nausea and vomiting. *N Engl J Med.* 2004;350:2441-2451.

**29.** Wongsurakiat P, Maranetra KN, Wasi C, Kositanont U, Dejsomritrutai W, Charoenratanakul S. Acute respiratory illness in patients with COPD and the effectiveness of influenza vaccination: a randomized controlled study. *Chest.* 2004;125:2011-2020.

**30.** Huskisson EC, Donnelly S. Hyaluronic acid in the treatment of osteoarthritis of the knee. *Rheumatology (Oxford).* 1999;38:602-607.

**31.** Adams ME, Atkinson MH, Lussier AJ, et al. The role of viscosupplementation with hylan G-F 20 (Synvisc) in the treatment of osteoarthritis of the knee: a Canadian multicenter trial comparing hylan G-F 20 alone, hylan G-F 20 with non-steroidal anti- inflammatory drugs (NSAIDs) and NSAIDs alone. *Osteoarthritis Cartilage.* 1995;3:213-225.

**32.** Khinchi MS, Poulsen LK, Carat F, Andre C, Hansen AB, Malling HJ. Clinical efficacy of sublingual and subcutaneous birch pollen allergen-specific immunotherapy: a randomized, placebo-controlled, double-blind, double-dummy study. *Allergy.* 2004;59:45-53.

**33.** James SK, Siegbahn A, Armstrong P, et al. Activation of the inflammation, coagulation, and fibrinolysis systems, without influence of abciximab infusion in patients with non-ST-elevation acute coronary syndromes treated with dalteparin: a GUSTO IV substudy. *Am Heart J.* 2004;147:267-274.

**34.** Rubin BR, Burton R, Navarra S, et al. Efficacy and safety profile of treatment with etoricoxib 120 mg once daily compared with indomethacin 50 mg three times daily in acute gout: a randomized controlled trial. *Arthritis Rheum.* 2004;50:598-606.

**35.** van Haselen RA, Fisher PA. A randomized controlled trial comparing topical piroxicam gel with a homeopathic gel in osteoarthritis of the knee. *Rheumatology (Oxford).* 2000;39:714-719.

**36.** Francis CW, Berkowitz SD, Comp PC, et al. Comparison of ximelagatran with warfarin for the prevention of venous thromboembolism after total knee replacement. *N Engl J Med.* 2003;349:1703-1712.

**37.** Kearon C, Ginsberg JS, Kovacs MJ, et al. Comparison of low-intensity warfarin therapy with conventional-intensity warfarin therapy for long-term prevention of recurrent venous thromboembolism. *N Engl J Med.* 2003;349:631-639.

**38.** Askie LM, Henderson-Smart DJ, Irwig L, Simpson JM. Oxygen-saturation targets and outcomes in extremely preterm infants. *N Engl J Med.* 2003;349:959-967.

**39.** Antoni C, Krueger GG, de Vlam K, et al. Infliximab improves signs and symptoms of psoriatic arthritis: results of the IMPACT 2 trial. *Ann Rheum Dis.* 2005.

**40.** Ridker PM, Goldhaber SZ, Danielson E, et al. Long-term, low-intensity warfarin therapy for the prevention of recurrent venous thromboembolism. *N Engl J Med.* 2003;348:1425-1434.

**41.** Crowther MA, Ginsberg JS, Julian J, et al. A comparison of two intensities of warfarin for the prevention of recurrent thrombosis in patients with the antiphospholipid antibody syndrome. *N Engl J Med.* 2003;349:1133-1138.

**42.** LaMonte MP, Nash ML, Wang DZ, et al. Argatroban anticoagulation in patients with acute ischemic stroke (ARGIS-1): a randomized, placebo-controlled safety study. *Stroke.* 2004;35:1677-1682.

**43.** Kearon C, Gent M, Hirsh J, et al. A comparison of three months of anticoagulation with extended anticoagulation for a first episode of idiopathic venous thromboembolism. *N Engl J Med.* 1999;340:901-907.

**44.** Konduri GG, Solimano A, Sokol GM, et al. A randomized trial of early versus standard inhaled nitric oxide therapy in term and near-term newborn infants with hypoxic respiratory failure. *Pediatrics.* 2004;113:559-564.

**45.** Coppen A, Noguera R, Bailey J, et al. Prophylactic lithium in affective disorders. Controlled trial. *Lancet.* 1971;2:275-279.

**46.** Prochazka AV, Kick S, Steinbrunn C, Miyoshi T, Fryer GE. A randomized trial of nortriptyline combined with transdermal nicotine for smoking cessation. *Arch Intern Med.* 2004;164:2229-2233.

**47.** Turner JA, Jensen MP, Warms CA, Cardenas DD. Blinding effectiveness and association of pretreatment expectations with pain improvement in a double-blind randomized controlled trial. *Pain.* 2002;99:91-99.

**48.** Hulley S, Grady D, Bush T, et al. Randomized trial of estrogen plus progestin for secondary prevention of coronary heart disease in postmenopausal women. Heart and Estrogen/progestin Replacement Study (HERS) Research Group. *Jama.* 1998;280:605-613.

**49.** Zajicek J, Fox P, Sanders H, et al. Cannabinoids for treatment of spasticity and other symptoms related to multiple sclerosis (CAMS study): multicentre randomised placebo-controlled trial. *Lancet.* 2003;362:1517-1526.

**50.** Donta ST, Engel CC, Jr., Collins JF, et al. Benefits and harms of doxycycline treatment for Gulf War veterans' illnesses: a randomized, double-blind, placebo-controlled trial. *Ann Intern Med.* 2004;141:85-94.

**51.** Carr A, Workman C, Carey D, et al. No effect of rosiglitazone for treatment of HIV-1 lipoatrophy: randomised, double-blind, placebo-controlled trial. *Lancet.* 2004;363:429-438.

**52.** Reinhart K, Gluck T, Ligtenberg J, et al. CD14 receptor occupancy in severe sepsis: results of a phase I clinical trial with a recombinant chimeric CD14 monoclonal antibody (IC14). *Crit Care Med.* 2004;32:1100-1108.

**53.** Leddy JJ, Epstein LH, Jaroni JL, et al. Influence of methylphenidate on eating in obese men. *Obes Res.* 2004;12:224-232.

**54.** Scognamiglio R, Negut C, Piccolotto R, Dioguardi FS, Tiengo A, Avogaro A. Effects of oral amino acid supplementation on myocardial function in patients with type 2 diabetes mellitus. *Am Heart J.* 2004;147:1106-1112.

**55.** Sutter FK, Simpson JM, Gillies MC. Intravitreal triamcinolone for diabetic macular edema that persists after laser treatment: three-month efficacy and safety results of a prospective, randomized, double-masked, placebo-controlled clinical trial. *Ophthalmology.* 2004;111:2044-2049.

**56.** Damico KE, Stoll AL, Marangell LB, Cohen BM. How blind is double-blind? A study of fish oil versus placebo. *Prostaglandins Leukot Essent Fatty Acids.* 2002;66:393-395.

**57.** Nanda A, O'Connor M, Anand M, et al. Dose dependence and time course of the immunologic response to administration of standardized cat allergen extract. *J Allergy Clin Immunol.* 2004;114:1339-1344.

**58.** Maestrelli P, Zanolla L, Pozzan M, Fabbri LM. Effect of specific immunotherapy added to pharmacologic treatment and allergen avoidance in asthmatic patients allergic to house dust mite. *J Allergy Clin Immunol.* 2004;113:643-649.

**59.** Robinson LR, Czerniecki JM, Ehde DM, et al. Trial of amitriptyline for relief of pain in amputees: results of a randomized controlled study. *Arch Phys Med Rehabil.* 2004;85:1-6.

**60.** Rorarius MG, Mennander S, Suominen P, et al. Gabapentin for the prevention of postoperative pain after vaginal hysterectomy. *Pain.* 2004;110:175-181.

**61.** Edwards JC, Szczepanski L, Szechinski J, et al. Efficacy of B-cell-targeted therapy with rituximab in patients with rheumatoid arthritis. *N Engl J Med.* 2004;350:2572-2581.

**62.** Buchbinder R, Hoving JL, Green S, Hall S, Forbes A, Nash P. Short course prednisolone for adhesive capsulitis (frozen shoulder or stiff painful shoulder): a randomised, double blind, placebo controlled trial. *Ann Rheum Dis.* 2004;63:1460-1469.

**63.** Khan Y, Heckmatt JZ. A double blind cross over trial of theophylline prophylaxis for sleep hypoxaemia in Duchenne muscular dystrophy. *Neuromuscul Disord.* 1997;7:75-80.

**64.** Shin DH, Feldman RM, Sheu WP. Efficacy and safety of the fixed combinations latanoprost/timolol versus dorzolamide/timolol in patients with elevated intraocular pressure. *Ophthalmology.* 2004;111:276-282.

**65.** Grigor C, Capell H, Stirling A, et al. Effect of a treatment strategy of tight control for rheumatoid arthritis (the TICORA study): a single-blind randomised controlled trial. *Lancet.* 2004;364:263-269.

**66.** Paganelli F, Cardillo JA, Melo LA, Jr., Oliveira AG, Skaf M, Costa RA. A single intraoperative sub-Tenon's capsule triamcinolone acetonide injection for the treatment of post-cataract surgery inflammation. *Ophthalmology.* 2004;111:2102-2108.

**67.** Ozolins M, Eady EA, Avery AJ, et al. Comparison of five antimicrobial regimens for treatment of mild to moderate inflammatory facial acne vulgaris in the community: randomised controlled trial. *Lancet.* 2004;364:2188-2195.

**68.** Brooks WA, Yunus M, Santosham M, et al. Zinc for severe pneumonia in very young children: double-blind placebo-controlled trial. *Lancet.* 2004;363:1683-1688.

**69.** Forster JG, Rosenberg PH. Small dose of clonidine mixed with low-dose ropivacaine and fentanyl for epidural analgesia after total knee arthroplasty. *Br J Anaesth.* 2004;93:670-677.

**70.** Spragg RG, Lewis JF, Walmrath HD, et al. Effect of recombinant surfactant protein C-based surfactant on the acute respiratory distress syndrome. *N Engl J Med.* 2004;351:884-892.

**71.** O'Brien L, Taddio A, Ipp M, Goldbach M, Koren G. Topical 4% amethocaine gel reduces the pain of subcutaneous measles-mumps-rubella vaccination. *Pediatrics.* 2004;114:e720-724.

**72.** Constant I, Leport Y, Richard P, Moutard ML, Murat I. Agitation and changes of Bispectral Index and electroencephalographic-derived variables during sevoflurane induction in children: clonidine premedication reduces agitation compared with midazolam. *Br J Anaesth.* 2004;92:504-511.

**73.** Bellack AS, Schooler NR, Marder SR, Kane JM, Brown CH, Yang Y. Do clozapine and risperidone affect social competence and problem solving? *Am J Psychiatry.* 2004;161:364-367.

**74.** Ascher B, Zakine B, Kestemont P, Baspeyras M, Bougara A, Santini J. A multicenter, randomized, double-blind, placebo-controlled study of efficacy and safety of 3 doses of botulinum toxin A in the treatment of glabellar lines. *J Am Acad Dermatol.* 2004;51:223-233.

**75.** Launay O, Roudiere L, Boukli N, et al. Assessment of cetirizine, an antihistamine, to prevent cutaneous reactions to nevirapine therapy: results of the viramune-zyrtec double-blind, placebo-controlled trial. *Clin Infect Dis.* 2004;38:e66-72.

**76.** McCarey BE, Kapik BM, Kane FE. Low incidence of iris pigmentation and eyelash changes in 2 randomized clinical trials with unoprostone isopropyl 0.15%. *Ophthalmology.* 2004;111:1480-1488.

**77.** Budde K, Curtis J, Knoll G, et al. Enteric-coated mycophenolate sodium can be safely administered in maintenance renal transplant patients: results of a 1-year study. *Am J Transplant.* 2004;4:237-243.

**78.** Filipponi F, Callea F, Salizzoni M, et al. Double-blind comparison of hepatitis C histological recurrence Rate in HCV+ Liver transplant recipients given basiliximab + steroids or basiliximab + placebo, in addition to cyclosporine and azathioprine. *Transplantation.* 2004;78:1488-1495.

**79.** O'Connor PW, Goodman A, Willmer-Hulme AJ, et al. Randomized multicenter trial of natalizumab in acute MS relapses: clinical and MRI effects. *Neurology.* 2004;62:2038-2043.

**80.** Scanlon PD, Connett JE, Wise RA, et al. Loss of bone density with inhaled triamcinolone in Lung Health Study II. *Am J Respir Crit Care Med.* 2004;170:1302-1309.

**81.** Holmes DR, Jr., Leon MB, Moses JW, et al. Analysis of 1-year clinical outcomes in the SIRIUS trial: a randomized trial of a sirolimus-eluting stent versus a standard stent in patients at high risk for coronary restenosis. *Circulation.* 2004;109:634-640.

**82.** Moller JE, Dahlstrom U, Gotzsche O, et al. Effects of losartan and captopril on left ventricular systolic and diastolic function after acute myocardial infarction: results of the Optimal Trial in Myocardial Infarction with Angiotensin II Antagonist Losartan (OPTIMAAL) echocardiographic substudy. *Am Heart J.* 2004;147:494-501.

**83.** Listrat V, Ayral X, Patarnello F, et al. Arthroscopic evaluation of potential structure modifying activity of hyaluronan (Hyalgan) in osteoarthritis of the knee. *Osteoarthritis Cartilage.* 1997;5:153-160.

**84.** Fiorucci S, Mencarelli A, Meneguzzi A, et al. Co-administration of nitric oxide-aspirin (NCX-4016) and aspirin prevents platelet and monocyte activation and protects against gastric damage induced by aspirin in humans. *J Am Coll Cardiol.* 2004;44:635-641.

**85.** Wiegman A, Hutten BA, de Groot E, et al. Efficacy and safety of statin therapy in children with familial hypercholesterolemia: a randomized controlled trial. *Jama.* 2004;292:331-337.

**86.** Sliwa K, Woodiwiss A, Kone VN, et al. Therapy of ischemic cardiomyopathy with the immunomodulating agent pentoxifylline: results of a randomized study. *Circulation.* 2004;109:750-755.

**87.** Valdivia I, Campodonico I, Tapia A, Capetillo M, Espinoza A, Lavin P. Effects of tibolone and continuous combined hormone therapy on mammographic breast density and breast histochemical markers in postmenopausal women. *Fertil Steril.* 2004;81:617-623.

**88.** Paya C, Humar A, Dominguez E, et al. Efficacy and safety of valganciclovir vs. oral ganciclovir for prevention of cytomegalovirus disease in solid organ transplant recipients. *Am J Transplant.* 2004;4:611-620.

**89.** Osman A, Otero J, Brizolara A, et al. Effect of rosiglitazone on restenosis after coronary stenting in patients with type 2 diabetes. *Am Heart J.* 2004;147:e23.

**90.** Panacek EA, Marshall JC, Albertson TE, et al. Efficacy and safety of the monoclonal anti-tumor necrosis factor antibody F(ab')2 fragment afelimomab in patients with severe sepsis and elevated interleukin-6 levels. *Crit Care Med.* 2004;32:2173-2182.
